# HLA and Non-HLA gene polymorphisms in autoimmune hepatitis patients of North Indian adults

**DOI:** 10.3389/fimmu.2022.984083

**Published:** 2023-01-18

**Authors:** Nishtha Ahuja, Jagdeep Singh, Ranjana Walker Minz, Shashi Anand, Ashim Das, Sunil Taneja

**Affiliations:** ^1^ Department of Histopathology, Postgraduate Institute of Medical Education and Research, Chandigarh, India; ^2^ Department of Immunopathology, Postgraduate Institute of Medical Education and Research, Chandigarh, India; ^3^ Department of Hepatology, Postgraduate Institute of Medical Education and Research, Chandigarh, India

**Keywords:** autoimmune hepatitis, adults, HLA, CTLA-4, PTPN22, polymorphisms, SLA

## Abstract

Autoimmune hepatitis (AIH) is a chronic and progressive disease of the liver. This is a multifactorial autoimmune disease with both environmental factors and genetic factors playing a role in its pathogenesis. Certain environmental agents like viruses, drugs, etc., can trigger the disease in a genetically susceptible individual. The present study was aimed to explore the distribution of human leukocyte antigen (HLA)-DRB1, Protein tyrosine phosphatase non-receptor type 22 (PTPN22) and Cytotoxic T-Lymphocyte-associated protein 4 (CTLA-4) polymorphisms in North Indian adult AIH patients and their associations with clinical and pathological characteristics associated with the disease. A total of 147 subjects with 47 cases and 100 healthy controls were enrolled. Diagnosis of AIH was made by Revised International Autoimmune Hepatitis Group scoring system. HLA-DRB1 Typing was done by Luminex-based reverse Sequence-Specific Oligonucleotide Probing (SSOP). Single nucleotide variant (SNV) genotyping for CTLA-4 and PTPN22 was done by simple probe-based SNP arrays. Results indicated SLA positive AIH patients are poor responders to therapy. A significant predispositional association of HLA-DRB1*03 was observed in AIH patients from the North Indian population (p= 0.0001, OR=4.83 (2.30-10.15). The frequency of the GG genotype of CTLA-4 CT 60 was significantly increased in AIH patients compared to controls. Multinomial analysis showed that CTLA-4 CT 60 is an independent predictor for cases.

## Introduction

Autoimmune hepatitis is a chronic and progressive disease of the liver. Patients present with varied symptoms ranging from asymptomatic to vague symptoms like arthralgia and/or fatigue to acute hepatitis-like presentation. Thus, it is often misdiagnosed initially. In India, all the centres do not have the facility for serological autoimmune workup, and around one-third of the patients have liver cirrhosis at the time of diagnosis (1). AIH patients respond to immunosuppression, emphasizing the importance of its early diagnosis and understanding its immunopathogenesis.

This is a multifactorial autoimmune disease with environmental and genetic factors playing a role in its pathogenesis. Certain environmental agents like viruses, drugs, etc., can trigger the disease in a genetically susceptible individual. Susceptibility to type 1 AIH (AIH-1), which is characterized by anti-nuclear (ANA) and/or smooth muscle antibodies (SMA), has been linked to MHC class II human leukocyte antigen (HLA) DRB1 alleles and an evident ethnic variation is seen in these associations (2–4). The structure of the surface binding groove of the class-II MHC molecule is determined by the HLA allele, which confers susceptibility to certain diseases. Different alleles of HLA play a role as a risk factor or a protective factor in different individuals. The HLA-DRB1 alleles HLA-DRB1*03:01, HLA-DRB1*04:01, HLA-DRB1*04:04 and HLA-DRB1*04:05 confer susceptibility to develop AIH-1 in different populations. These alleles express proteins which have similar 6 amino acid sequences (LLEQR or LLEQRR) at position 67 to 72 in the antigen-binding groove of Class-II MHC molecule (5). Among Asians, different studies have found an association between HLA-DR4 in East Asian and HLA-DR3 in South Asian populations with AIH-1 (6).

Cytotoxic T lymphocyte-associated protein 4 *(*CTLA-4*)*, a widely studied non-HLA susceptibility gene in autoimmune disorders, is mainly expressed on the surface of regulatory T cells and conventional T cells and suppresses self-reactive T cells responses *via* downregulating ligand availability for the costimulatory receptor CD28 to elicit inhibitory signals. Single nucleotide variant (SNV) in CTLA-4 CT60 +6230G/A has been shown to be associated with reduced sCTLA-4 and higher risk for acute rejection in allogenic liver transplant patients (7). While another non-HLA gene, Protein tyrosine phosphatase non-receptor type 22 (*PTPN22*), encodes the protein lymphoid tyrosine phosphatase (LYP), which regulates the activation of protein kinases to modulate intracellular tyrosine phosphorylation incidence and act as a regulator of molecular signal transduction (8). PTPN22 -C1858T is the most studied SNV in the field of autoimmunity (9).

Since AIH is a rare disease and it is quite challenging to enlarge the sample size for the Genome Wide Association Study (GWAS). There has been only one GWAS aimed at AIH in the literature till now. This GWAS, performed on the European population, demonstrate significant associations of SNVs in HLA and some of the non-HLA genes. The three main non-HLA genes in concern were SH2B3 (Src Homology 2 Adapter Protein 3), CARD10 (Caspase Recruitment Domain Family member 10) and ICOS (Inducible co-stimulator). The other genes, such as STAT4, IL-12A, IL-12RB, BACH2 and CTLA-4/CD28, were described as nominally significant (2). After this GWAS, several major attempts have been made to delineate the genetic architecture and its contribution to disease pathogenesis (3, 4). However, only a limited number of studies have explored the non-HLA association with AIH.

From India, there is no study regarding the non-HLA gene associations with the disease, and only a few studies have demonstrated the HLA-DRB1 association in the same disease. Within the same country, studies have shown variability in the association between HLA alleles and AIH (10, 11). Therefore, we conducted a case-control association study examining eight non-HLA candidate loci (3 SNVs for PTPN22 rs2476601, rs1217412, rs2488457 and 5 for CTLA-4 rs3087243, rs231775, rs5742909, rs4553808 and rs733618) in north Indian adult AIH patients. We also performed an HLA-DRB1 association analysis in these patients. We compared HLA and non-HLA associations with disease outcome and treatment response and also compared cases with or without Anti-SLA antibodies (Antibodies against soluble liver antigen/liver-pancreas) with treatment outcomes and if any of the genetic components correlated with the clinical parameters of the disease.

## Materials and methods

### Study population

A total of 147 subjects were recruited, with 47 cases and 100 healthy controls. Patients were recruited from the Department of Hepatology of PGIMER, Chandigarh. Diagnosis of AIH was made by Revised International Autoimmune Hepatitis group scoring system for diagnosis of AIH (12). Patients diagnosed with type-2 AIH who were positive for LKM1 and/or LC1, AIH-PSC overlap, AIH-Autoimmune sclerosing cholangitis and AIH-PBC overlap were excluded from the study.

The controls were age, gender, and ethnicity matched with the patients and were not suffering from any disease. A written informed consent was obtained from all the participants after explaining the aims of the study. The study was approved by institutional ethics committee of PGIMER (INT/IEC/2018/000795). Clinical details were taken from the patients and their Liver clinic outpatient record files.

### Sample collection and autoantibody screening

Five millilitres (ml) of peripheral venous blood sample was obtained from the patients and was divided into two portions: 3 ml in plain vials for serum separation and 2 ml in EDTA vials for DNA extraction. AMA, SMA, ANA, LKM and LC-1 were detected in the serum of patients by indirect immunofluorescence (IIF) and confirmed by Immunoblot (Euroimmune) and ELISA except for SMA. SLA ELISA was performed using QUANTA Lite^®^ SLA ELISA kit by Inova Diagnostics. Patients showing positivity for Anti-LKM/Anti-LC-1 antibody were excluded from our study. We had excluded two such patients from our study (positive for Anti-LKM antibody) as there is different genetic susceptibility for AIH-1 and AIH-2, thus we had enrolled adult Non-type 2 AIH cases (13).

### DNA extraction, HLA and non-HLA genotyping

DNA extraction was done using spin column-based method (QIAmp DNA, Qiagen). HLA-DRB1 typing was done by Luminex-based reverse Sequence-Specific Oligonucleotide Probing (SSOP) (for details, see **Supplementary File S1**). Due to constraint resources, we had performed low-resolution HLA typing.

SNV genotyping was done by simple probe-based SNP arrays (Roche Diagnostics) for the following genes - CTLA-4 [+49 A/G (rs 231775), -318 C/T (rs 5742909), CT 60 (+6230G/A, rs3087243), -1722 C/T (rs 733618) and -1661 A/G (rs 4553808)] and PTPN22 [+2740 A/G (rs 1217412), -1123 C/G/T (rs 2488457) and +1858 C/T (rs 2476601)] (for details see **Supplementary File S1**). We had chosen these 8 SNPs based on the GWAS on AIH and other autoimmune diseases. Both of them are negative regulators of T cells and present knowledge underlines the understanding that polymorphisms in these two genes are associated with loss of tolerance, autoimmunity and also susceptibility to cancers (2–4).

### Statistical analysis

Data are expressed as mean with standard deviation and median with interquartile range, as applicable. Allelic and genotypic frequencies were compared between patients and controls using χ2 test with Yates’ correction. Calculations of odds ratios (ORs) and 95% confidence intervals (CIs) for relative risks were performed after the application of Fisher’s exact test, if appropriate. Two-sided *P* values less than 0.05 were considered significant. The analysis was conducted using IBM SPSS STATISTICS (version 22.0).

## Results

### Clinical characteristics of the study Participants

**Figure 1:**
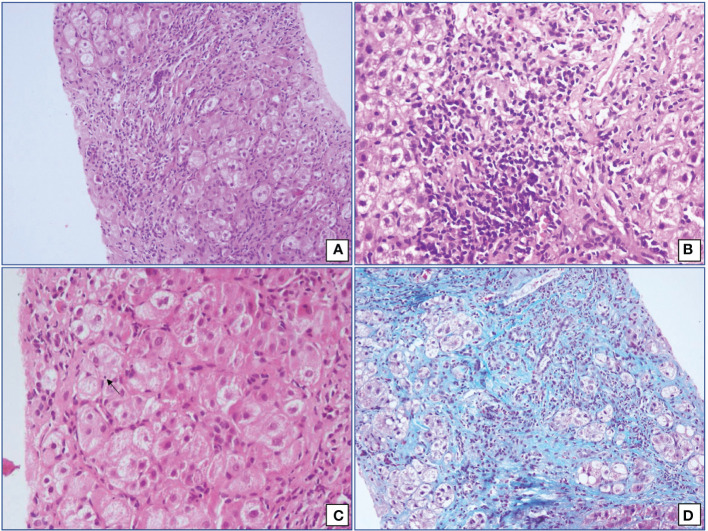
Microphotographs from cases of Autoimmune hepatitis showing **(A)** interface hepatitis with expanded portal tracts and significant portal tract inflammation (Hematoxylin and Eosin; 200x), **(B)** moderate lymphoplasmacytic cells infiltrate in an irregular and expanded portal tract (Hematoxylin and Eosin; 400x), **(C)** emperipolesis (Hematoxylin and Eosin; 400x) and **(D)** hepatocyte rosetting (Masson’s Trichrome; 200x).

There was a female preponderance with female: male ratio of 2.6:1. Patients had a variable presentation of the disease with a mean age at the time of presentation 32.82 years (SD ±13.6). One-third of our patients (n=16) had acute presentation (Acute presentation was categorized as a cut-off of bilirubin >2 mg/dl and ALT or AST levels >10 times the Upper Limit of Normal) (14). The baseline characteristics of AIH patients are given in **Table 1**. Histopathological assessment of liver biopsy was done in 41 cases. The characteristic histopathological features are shown in **Figure 1**. In all the 41 cases, significant interface hepatitis was present; in 29 cases, lymphoplasmacytic infiltration in the portal tracts was seen, 25 cases had hepatocyte rosetting, and 3 cases had emperipolesis. None of the cases showed any biliary changes. 42.55% (n=20) of the patients had liver cirrhosis at the time of presentation at our institute. In the cases where liver biopsy was not available or was inadequate for opinion, Fibroscan (Transient Elastography) reports were considered for the status of fibrosis. A cut-off of 12.67 kPa was taken for Cirrhosis (15). The liver biopsy fibrosis scoring was done by the French Metavir scoring system (16). 42.5% (n=20) of our patients had concurrent other autoimmune diseases, with the most common being Hashimotos thyroiditis (n=11, 23.4%) followed by Celiac disease (n=2, 4.25%). The patients of Hashimotos thyroiditis had raised levels of serum TSH and Anti-TPO antibodies at the time of diagnosis.

**Table 1:** Baseline characteristics of AIH-1 patients.

Parameters	
Age (Years)	36.56 ± 13.50 (Mean ± SD)
Female: Male	2.6:1
Time Of Presentation (Age In Years)	36.34 ± 13.4 (Mean ± SD)
Acute presentation of disease	n= 16 (34.04%)
Cirrhosis*	n= 20 (42.5%)
AST (U/L)	166 (86-501) Median (IQR)
ALT (U/L)	150.6 (72-342) Median (IQR)
ALP (U/L)	192 (135.3-264) Median (IQR)
Total Bilirubin (mg/dL)	2.8 (1.17-5.50) Median (IQR)
Total Protein	7.60 (6.70-8.8) Median (IQR)
Albumin	3.6 (2.99-4.06) Median (IQR)
Serum IgG	
WNL	n= 9 (19.14%)
Raised	n= 38 (80.85%)
Serum IgG (times of Upper normal limit)	1.43 (1.24-1.87) Median (IQR)
Serum profile	
ANA	n= 18 (38.3%)
SMA	n= 21 (44.7%)
ANA & SMA	n= 3 (6.3%)
SLA	n= 7 (14.9%)
SLA & ANA	n= 1 (2.1%)
SLA & SMA	n= 4 (8.5%)
Seronegative	n= 9 (19.15%)
Other Autoimmune diseases	n= 20 (42.5%)
Liver biopsy histopathological findings	
Significant interface hepatitis	n= 41 (87.23%)
Lymphoplasmacytic infiltration	n= 29 (61.7%)
Hepatocyte resetting	n= 26 (55.32%)
Emperipolesis	n= 3 (6.4%)
Diagnosis (Revised IAIHG)	
Probable AIH	n= 11 (23.4%)
Definite AIH	n= 36 (76.59%)
Response To Treatment**	
Complete Response	n= 25 (53.2%)
Incomplete Response	n= 16 (34.04%)
Relapse	n= 5 (10.6%)

* In two cases both fibroscan and liver biopsy were not available, **one case was lost to follow-up.

AST, Aspartate Aminotransferase; ALT, Alanine Aminotransferase; U/L, IQR, Interquartile Range; SD, Standard Deviation; IAIHG, International Autoimmune Hepatitis Group; AIH, Autoimmune Hepatitis; WNL, Within Normal Limit.

All the patients were given immunosuppression; n=25 (53.2%) showed complete response, n=16 (34%) showed an incomplete response and n=5 (10.6%) showed relapse after discontinuation of therapy. Thirty seven cases (78.72%) were reactive for autoantibodies, namely ANA and/or SMA and/or SLA. Nine patients (19.15%) were seronegative. Among the nine seronegative patients, 4 were diagnosed as definite AIH and rest of the 5 patients were diagnosed as probable AIH as per Revised IAIHG criteria. Majority of these patients had shown complete response to immunosuppression (n=7, 77.7%) with only two cases with incomplete response and none with relapse. Two out of these nine patients had cirrhosis at the time of presentation in our institute. One patient was a female who presented at the age of 58 years with insidious onset. Her serum IgG levels were raised (1.43 times the upper normal limit), her liver biopsy showed significant interface hepatitis along with F4 fibrosis (Metavir scoring system) and she showed complete response on immunosuppression. She was heterozygous for HLA-DRB1*03 and had associated Hypothyroidism. The other patient was a 18 years old male who also presented with insidious onset. He had raised serum IgG levels (1.8 times the upper normal limit) and his liver biopsy showed significant interface hepatitis and moderate lymphoplasmacytic inflammation in the portal tracts along with F4 fibrosis (Metavir scoring system). He was also heterozygous for HLA-DRB1*03. However, he showed incomplete response on immunosupression and was advised for liver transplantation. There was no history of alcohol intake or hepatotoxic drug intake in both of these patients and viral markers were negative.

We observed that Anti-SLA ELISA positive cases were poor responders to immunosuppressive therapy, with no case showing complete response compared to 62.5% in cases without Anti-SLA autoantibodies (χ2 = 8.97, p = 0.004) (**Table 2**). Strength of association between the two variables (Cramer’s V) = 0.442 (Relatively strong Association). Strength of association between the two variables (Bias Corrected Cramer’s V) = 0.392 (Moderate Association).

**Table 2:** Response to treatment in Anti-SLA ELISA positive cases.

Response to treatment	SLA ELISA		P value
	Positive (n=7)*	Negative (n=40)	
Complete Response	0 (0.0%)	25 (62.5%)	0.004
Incomplete Response	4 (66.7%)	12 (30.0%)	
Relapse	2 (33.3%)	3 (7.5%)	

*One patient was lost to follow-up.

SLA, Antibodies to Soluble Liver Antigen/liver-pancreas; ELISA, Enzyme-Linked Immunoassay.

### HLA-DRB1 allele susceptibility

The allele frequency of DRB1*03 was significantly higher in AIH patients as compared to healthy controls (P = 0.0001, OR = 4.83, 95% CI = 2.30 – 10.15, P-value with Bonferroni correction [Pc] = 0.001). The frequencies of the alleles obtained in the patients and controls are shown in **Table 3**. We also tested by discriminating allele frequencies in different sub-group of patients, categorized on the basis of their clinical features, presence of cirrhosis and response to immunosuppression. However, no significant association was observed between HLA-DRB1 and different categories of the patients.

**Table 3:** Genotype frequency of HLA DRB1 in cases and controls.

HLA DRB1	AIH Patients n=47	Healthy Controls n= 100	P value	Pc value	Odds Ratio (95% CI)	Relative Risk (95% CI)
DRB1*01	1 (2.1%)	11 (11%)	0.131	1.703	0.18 (0.02-1.40)	0.24 (0.03-1.62)
DRB1*03	29 (61.7%)	25 (255%)	0.0001	0.001	4.83 (2.30-10.15)	2.78 (1.71-4.50)
DRB1*04	3 (6.4%)	12 (12%)	0.449	5.837	0.50 (0.13-1.87)	0.60 (0.21-1.70)
DRB1*07	10 (21.3%)	22 (22%)	0.908	11.80	0.96 (0.41-2.23)	0.97 (0.55-1.73)
DRB1*08	0 (0.0%)	2 (2%)	0.831	10.80	0.42 (0.02-8.82)	0 (Infinity)
DRB1*09	0 (0.0%)	0 (0.0%)	**-**	**-**	**-**	**-**
DRB1*10	1 (2.1%)	11 (11%)	0.131	1.703	0.18 (0.02-1.41)	0.25 (0.04-1.62)
DRB1*11	4 (8.5%)	19 (19%)	0.165	2.145	0.40 (0.13-1.24)	0.50 (0.20-1.26)
DRB1*12	0 (0.0%)	0 (0.0%)	**-**	–	**-**	**-**
DRB1*13	9 (19.1%)	18 (18%)	0.952	12.38	1.08 (0.44-2.62)	1.05 (0.58-1.91)
DRB1*14	7 (14.9%)	21 (21%)	0.513	6.67	0.66 (0.26-1.68)	0.74 (0.37-1.48)
DRB1*15	19 (40.4%)	38 (38%)	0.920	11.96	1.11 (0.55-2.25)	1.07 (0.66-1.73)
DRB1*16	1 (2.1%)	2 (2%)	0.566	7.36	1.07 (0.09-12.06)	1.04 (0.21-5.26)

### Non-HLA association

SNP arrays were done for 5 SNVs in CTLA-4 and 3 SNVs in PTPN22. The genotype frequencies are given in **Table 4**. There was a significant difference between the cases and controls in terms of distribution of CTLA-4 CT 60: A/G (p = 0.0003, OR = 5.1, 95% CI = 2.107-12.34). GG genotype of CTLA-4 CT 60 was significantly higher in cases (36.2%) as compared to controls (10.0%). Strength of association between the two variables (Cramer’s V) = 0.341 (Moderate Association). Strength of association between the two variables (Bias Corrected Cramer’s V) = 0.322 (Moderate Association). On dividing cases based on their gender, GG genotype showed marked susceptibility for AIH while GA genotype was protective, especially in females. There was no significant association of all the 8 SNVs with reference to age at presentation, clinical features at the time of presentation, presence or absence of cirrhosis and response to immunosuppressive therapy and no significant association between HLA-DRB1 typing of all the cases and all the eight SNVs was obtained.

**Table 4:** Genotype and allele frequencies of SNVs of CTLA-4 and PTPN22 in cases and controls.

Genotype	AIH Patients (n=47)	Healthy Controls (n= 100)	p value OR (95% CI)
CTLA-4 CT60 (rs 3087243)			
AA	16 (34.0%)	32 (32.0%)	p=0.954 OR=1.097 (0.525-2.288)
GA	14 (29.8%)	58 (58.0%)	p=0.002 OR=0.307 (0.146-0.644)
GG	17 (36.2%)	10 (10.0%)	p=.0003 OR=5.100 (2.107-12.34)
A	46 (48.9%)	122 (61.0%)	p=0.068 OR=0.612 (0.373-1.005)
G	48 (51.1%)	78 (39.0%)	p=0.068 OR=1.632 (0.995-2.676)
CTLA-4 49 (rs 231775)			
AA	22 (46.8%)	47 (47.0%)	p=0.876 OR=0.994 (0.620-1.596)
AG	18 (38.3%)	45 (45.0%)	p=0.557 OR=0.758 (0.373-1.540)
GG	7 (14.9%)	8 (8.0%)	p=0.319 OR=2.013 (0.683-5.929)
A	62 (66.0%)	139 (69.5%)	p=0.635 OR=0.850 (0.504-1.433)
G	32 (34.0%)	61 (30.5%)	p=0.635 OR=1.176 (0.697-1.983)
CTLA-4 318 (rs 5742909)			
CC	41 (87.2%)	92 (92.0%)	p=0.537 OR=0.594 (0.193-1.823)
CT	6 (12.8%)	8 (8.0%)	p=0.537 OR=1.683 (0.548-5.163)
C	88 (93.6%)	192 (96.0%)	p=0.547 OR=0.611 (0.205-1.815)
T	6 (6.4%)	8 (4.0%)	p=0.547 OR=1.636 (0.551-4.859)
CTLA-4 1661 (rs4553808)			
AA	39 (83.0%)	77 (77.0%)	p=0.540 OR=1.456 (0.646-3.554)
AG	8 (17.0%)	14 (14.0%)	p=0.817 OR=1.260 (0.488-3.251)
GG	0 (0.0%)	9 (9.0%)	p=0.033 OR=0.101 (0.005-1.781)
A	86 (91.5%)	168 (84.0)	p=0.117 OR=2.048 (0.904-4.637)
G	8 (8.5%)	32 (16.0%)	p=0.117 OR=0.488 (0.215-1.106)
CTLA-4 1722 (rs733618)			
AA	33 (70.2%)	79 (79.0%)	p=0.337 OR=0.626 (0.284-1.379)
AG	11 (23.4%)	20 (20.0%)	p=0.798 OR=1.222 (0.530-2.816)
GG	3 (6.4%)	1 (1.0%)	p=0.184 OR=6.750 (0.682-66.75)
A	77 (81.9%)	178 (89.0%)	p=0.137 OR=0.559 (0.281-1.113)
G	17 (18.1%)	22 (11.0%)	p=0.137 OR=1.786 (0.898-3.552)
PTPN22 1858 (rs 2476601)			
AA	4 (8.5%)	5 (5.0%)	p=0.646 OR=1.767 (0.452-6.911)
GA	1 (2.1%)	0 (0.0%)	p=0.319 OR=6.484 (2.590-162.3)
GG	42 (89.4%)	95 (95.0%)	p=0.360 OR=0.442 (0.121-1.609)
A	9 (9.6%)	10 (5.0%)	p=0.217 OR=2.012 (0.788-5.132)
G	85 (90.4%)	190 (95.0%)	p=0.217 OR=0.497 (0.194-1.268)
PTPN22 2740 (rs 1217412)			
AA	28 (59.6%)	53 (53.0%)	p=0.568 OR=1.307 (0.647-2.639)
AG	16 (34.0%)	45 (45.0%)	p=0.281 OR=0.630 (0.306-1.297)
GG	3 (6.4%)	2 (2.0%)	p=0.379 OR=3.341 (0.538-20.72)
A	72 (76.6%)	151 (75.5%)	p=0.953 OR=1.062 (0.596-1.890)
G	22 (23.4%)	49 (24.5%)	p=0.953 OR=0.941 (0.529-1.675)
PTPN22 1123 (rs 2488457)			
CC	2 (4.3%)	8 (8.0%)	p=0.624 OR=0.511 (0.104-2.507)
GC	14 (29.8%)	34 (34.0%)	p=0.749 OR=0.823 (0.389-1.743)
GG	31 (65.9%)	58 (58.0%)	p=0.459 OR=1.403 (0.681-2.890)
C	18 (19.1%)	50 (25.0%)	p=0.301 OR=0.710 (0.387-1.302)
G	76 (80.9%)	150 (75.0%)	p=0.301 OR=1.407 (0.768-2.579)

CTLA-4, Cytotoxic T-Lymphocyte Antigen-4; PTP, Protein Tyrosine Phosphatases; SNV, Single Nucleotide Variant.

## Discussion

This study is from a tertiary referral centre in North India with an extended panel of screening for autoantibodies, including the AIH specific auto-antibodies against SLA. It is also well characterized with biopsy in 87.2% of patients.

One-third of our patients presented with acute hepatitis; these cases could be acute AIH or the acute exacerbation of AIH. Cirrhosis is more frequently observed in our cohort (43.5%) compared to studies on the Chinese population (17). However, different studies have observed that AIH patients of South Asian origin present late with cirrhosis and have higher mortality rates than patients of East Asian origin (6, 18). An extended serological workup was included, involving Anti-SLA ELISA and IIF for ANA, AMA, LKM and SMA autoantibodies.

Out of all cases, 19.15% (n=9) were seronegative, which is as described in the literature (19). We divided the cases into Anti-SLA positive and Anti-SLA negative groups and analyzed various parameters to see if they presented any different from each other. Anti-SLA positivity was seen in 14.9% (n=7) of patients. Autoantibodies against SLA have a high specificity (99%) for the diagnosis of AIH (20). Among 4.2% (n=2) of all the patients, Anti-SLA was the only antibody detected, which is much less as compared to previous reports (21). In 10.6% (n=5) of our patients, Anti-SLA positivity was present along with ANA/SMA positivity. After comparing Anti-SLA positive and Anti-SLA negative groups on various parameters with each other, we found that Anti-SLA positive cases showed significantly poor response to immunosuppression compared to Anti-SLA negative cases. Similarly, Ma Y et al. in a study had found that Anti-SLA positive autoimmune liver disease patients have a more severe disease clinical course, take longer time to respond to immunosuppression and have more incidences of relapse (22). On the other hand, Zhi-Xian Chen et al. demonstrated that treatment response was comparable between the groups, 72% and 71.3% in Anti-SLA positive and negative groups, respectively (23). Zhi-Xian Chen et al. conducted a meta-analysis and they also described a significant risk of relapse in the Anti-SLA positive groups (23). Zachou et al. also showed treatment relapse after corticosteroid withdrawal and thus proposed lifelong immunosuppression in these patients (24). Therefore, including SLA antibodies in screening panel can increase AIH detection in the population as well as help in their plan of treatment. In North India, the prevalence spectrum of autoimmune liver diseases is different from that described in Caucasian populations. Here, AIH is the most common autoimmune liver disease, followed by AIH/PBC overlap (25).

The distribution of HLA-DRB1 showed a positive association of DRB1*03 allele with AIH patients from India. This is in conjunction with the studies in Caucasian European and North American populations, where along with HLA-DRB1*03:01, HLA-DRB1*04:01 and HLA-DRB1*01:01 were also significantly associated. Even in the Brazilian population, HLA-DRB1*03:01 has a secondary association with the disease (26). Similarly, DRB1*03 is also a susceptible allele for various other autoimmune disorders such as autoimmune thyroiditis (27), PR3-ANCA-associated vasculitis (28), systemic lupus erythematosus, multiple sclerosis, and myasthenia gravis (29).

Previously Kaur et al., 2014 described HLA-DRB1*04 and HLA-DRB1*08 as susceptible alleles for AIH-1 in the North Indian population (11). It was also observed that HLA-DRB1*04 was significantly associated with pediatric AIH-1 patients, and DRB1*08 was associated with adult AIH-1 patients. The discordance of these results with our study can be explained by the inclusion of only adult patients in the present study, of which 87.2% were biopsy-proven. Additionally, the presence of SLA positivity is also demonstrated to be associated with HLA-DRB1*03 (30). Further salient reports on HLA association with AIH in different ethnic groups are illustrated in **Supplementary Table 1**.

The genetic complexity of autoimmune diseases has led to many research studies regarding the association of non-HLA genes. In AIH, associations of CARD10, SH2B3, and ICOS genes were suggested in GWAS by de Boer et al. in 2014 (2). Higuchi et al. in 2021 have enlisted the association of other genes, namely KIR, PTPN22, SH2B3, TNFAIP3, STAT4, TNIP1, CTLA-4, FAS and TNF (31) (**Supplementary Table 2**). We studied SNVs of CTLA-4 and PTPN22 genes. We had selected a larger number of polymorphisms in these two genes (a total number of 8 SNPs) to be able to report ethnic genetic variations in our cohort of AIH. The other reason for choosing these SNPs was that we had already studied these SNPs in ANCA associated vasculitis patients and thus had planned to look for the same SNPs in Autoimmune Hepatitis (28). From India, there has not been any study regarding non-HLA association gene polymorphisms with AIH. In this study, we found GG genotype of CTLA-4 CT 60 to be significantly associated with AIH patients (p=0.0003). In contrast, no significant difference in the genotype frequencies was observed in analyzing +49 A/G (rs 231775), -318 C/T (rs 5742909), -1722 C/T (rs 733618) and -1661 A/G (rs 4553808) polymorphisms of CTLA-4 gene. Agarwal et al. analyzed +49 A/G (rs 231775) polymorphism of the CTLA-4 gene and detected a high G allele frequency in patients compared to controls (32). They have described the association of GG genotype with greater mean AST levels and higher frequency of detection of Anti-TMA (Anti-Thyroid Microsomal Antigen) and HLA-DRB1*03:01. However, we could not find any relation between these polymorphisms and biochemical parameters, clinical signs and symptoms, treatment outcome and HLA-DRB1 typing.

Mutation in PTNP22 gene may increase susceptibility to various autoimmune diseases such as Type 1 Diabetes Mellitus, Rheumatoid Arthritis etc. A study in the Japanese population found a significant difference in minor allele frequencies of rs 1217412 in patients compared to controls (33). Li et al. analysed -1123 C/G/T (rs 2488457) and +1858 C/T (rs 2476601) polymorphisms in Chinese Han population and found that risk of AIH-1 was lower in carriers of T allele than CC genotype (CT + TT versus CC) in +1858 C/T (rs2476601), [OR (95% CI) = 0.65 (0.44–0.93)] (34). Similar to our results, Li et al. also didn’t find any association between -1123 C/G/T (rs 2488457) and AIH-1 risk. Moreover, earlier studies also have demonstrated the absence of +1858 C/T association with SLE (35), vitiligo (36) and ANCA-associated vasculitis (28) except for rheumatoid arthritis (37) and type 1 diabetes (38).

Analysis based on clinical categorization such as the presence of cirrhosis, response to treatment, increased serum IgG levels and concurrent other autoimmune disorders indicates no significant correlation with non-HLA genes. This was similar to results demonstrated in North European Caucasoid population (32). On the contrary, Fortes et al. confirmed the association of PTPN22 1858 C/T with cirrhosis, increased serum IgG levels and treatment response in Mestizo Venezuelan population (39). In the North European Caucasoid population, there was a synergy between CTLA-4 +49A/G and DRB1*03:01 in susceptibility to AIH-1 (32). However, we could not demonstrate any such association between them. The small sample size and low-resolution HLA typing are the major limitations of this study. This is a pilot study, and it needs to be validated in a larger cohort.

## Conclusion

In a rare disease like AIH, we were able to describe HLA and non-HLA polymorphisms in a fairly large single-centre study. HLA-DRB1*03 is the susceptibility allele, and CTLA-4 CT60 is an independent predictor of disease. AIH remains underdiagnosed in India and picked up in the late stage. Screening should be enhanced in the country with an extended AIH panel to diagnose SLA-positive cases as they portend resistance to therapy. In future, registries and multicentric collaboration throughout the country should aim at a GWAS from India. It could unravel ethnic variations in genetics as well as the phenotype of the disease.

## Data availability statement

Datasets used and/or analyzed during the current study are available from the corresponding author on reasonable request.

## Ethics statement

The study involving human participants was reviewed and approved by IEC, Postgraduate Institute of Medical Education and Research, Chandigarh. The patients/participants provided their written informed consent to participate in this study.

## Author contributions

NA: executed the study, analyzed the data, and wrote the manuscript. JS: contributed in execution of study, analyzed the data and helped in manuscript writing. RM: designed the study, analyzed the data, edited and corrected the manuscript. SA: contributed in execution of Real-Time PCR for SNV polymorphism. AD: reported and evaluated the histopathology of liver. ST: helped in recruitment of patients, treated the patients, and provided the clinical details of the patients. All authors contributed to the article and approved the submitted version.
