# Supplementary material for: HLA and Non-HLA gene polymorphisms in autoimmune hepatitis patients of North Indian adults
Source: Front Immunol. 2023 Jan 18;13:984083. doi: 10.3389/fimmu.2022.984083 (PMC9891307; doi:10.3389/fimmu.2022.984083)
Supplement: Supplementary file 1 [file Table_1.docx]

**Supplementary Table 1:** Reports on HLA genetic association with AIH-1 in different ethnic groups.

| **Study** | **Year** | **Population** | **Cases** | **Controls** | **HLA allele** |
| --- | --- | --- | --- | --- | --- |
| Strettell et al. (40) | 1997 | North Europe | 86 | 102 | **Risk-** HLA-A1-HLA-B8-DRB3*01:01-DRB1*03: 01-DQA1*05:01-DQBl*02:01 HLA-DRB1*04:01  **Protective-** HLA-DRB5*01:01-DRB1*15:01 |
| Yoshizawa et al. (41) | 2005 | Japanese | 77 | 248 | **Risk-** HLA-DR4*04:04 and HLA-DRB*04:05 |
| Umemura et al. (42) | 2014 | Japanese | 156 | 210 | **Risk-**DRB1*04:05-DQB1*04:01, HLA A*24:02 and C*01:02  **Protective-** DRB1*15:01-DQB1*06:02 |
| Furomoto et al. (43) | 2015 | Japanese | 132 | 31973 | **Risk-** HLA DR4 |
| Oka et al. (44) | 2017 | Japanese | 360 | 1026 | **Risk-** DRB1*04:01, DRB1*04:05 and DQB1*04:01  **Protective-**DRB1*13:02 |
| Bittencourt et al. (45) | 1999 | Latin America | 139 | 129 | **Risk-** DRB1*13:01-DQB1*06  **Protective-** HLA-DQB1*04-DQB1*03:01 |
| Duarte-Rey et al*.* (46) | 2009 | Latin American | 694 | 1075 | **Risk-** DQB1⁎02, DQB1⁎0603, DRB1⁎0405, and DRB1⁎1301  **Protective-** DRB1⁎1302 and DQB1⁎0301 |
| Amarapurkar et al. (10) | 2003 | West Indian | 20 | 100 | **Risk-** HLA-DRB1*01 and HLA-DRB1*14 HLA-DQB1*04-DQB1*03:01 |
| Kaur et al.  (11) | 2014 | North Indian | 55 | 128 | **Risk-**HLA DRB1*04 and DRB1*08 |
| Hassan et al. (47) | 2013 | Pakistani | 44 | 912 | **Risk-**HLA DRB1*13, HLA DRB1*14 |
| [Lim](https://pubmed.ncbi.nlm.nih.gov/?term=Lim+YS&cauthor_id=18022727) et al. (48) | 2008 | Korean | 62 | 154 | **Risk-**HLA DRB1*04:05, HLA DQB1 *0401 |
| [Qiu](https://pubmed.ncbi.nlm.nih.gov/?term=Qiu+DK&cauthor_id=12519226) et al. (49) | 2003 | Chinese | 32 | 48 | **Risk-** HLA DR4 |

**Supplementary Table 2:** Reports on non-HLA genetic association with AIH-1 in different ethnic groups.

| **Study** | **Year** | **Population** | **Cases** | **Controls** | **Gene** | **Non-HLA gene mutation** |
| --- | --- | --- | --- | --- | --- | --- |
| Webb et al. (50) | 2016 | European Caucasian ancestry | 1 | - | **GATA-2** | mutation |
| Sun et al. (51) | 2017 | Chinese | 20 | 35 | **TBX21** | **Protective -1993C** |
| Li et al. (34) | 2017 | Chinese, Japanese | 180 | 362 | **STAT 4** | **Risk-** rs7574865 and rs7582694 in minor alleles, interaction between rs7582694 and rs2476601, and haplotype containing the rs7582694-C and rs7574865-T alleles |
| Li et al. (34) | 2017 | Chinese, Japanese | 180 | 362 | **PTPN22** | **Protective -rs2476601** |
| Umemura et al. (33) | 2016 | Japanese | 166 | 322 | **PTPN22** | **Protective -minor alleles at rs1217412, rs1217388, rs1217407, and rs2488458; and AAGTCCC haplotype** |
| Umemura et al. (52) | 2017 | Japanese | 158 | 325 | **SH2B3** | **Protective -rs2238154 A, rs11065904 T and rs739496 G** |
| Littera et al. (53) | 2016 | Europeian (Sardinian) | 114 | 221 | **KIR** | **Risk-** KIR2DS1 |
| Agarwal et al. (54) | 2007 | Caucasoid, Northern European | 149 | 172 | **FAS (TNFRSF)** | *** |
| Kempinska-Podhorodecka et al. (55) | 2020 | European (Polish) | 142 | 376 | **VDR** | **Risk-** TT genotype of Taql (rs731236), AA genotype of Bsml (rs1544410) and AA genotype of Apal (rs7975232)  **Protective -**CC genotype of Taql (rs731236) |
| Chaouali et al. (56) | 2018 | North Africa (Tunisian) | 50 | 100 | **CTLA-4** | **Risk-** G genotype in +49 position |
| Higuchi et al. (57) | 2017 | Japanese | 343 | 315 | **ICOS** | **Risk-** G allele rs4325730 upstream of **ICOS** gene. |
| Assis et al. (58) | 2014 | United States | 52 | 286 | **MIF** | **Risk-** -794 CATT_7_  of **MIF** gene |
| Czaja et al. (59) | 1999 | European | 155 | 102 | **TNF-A** | **Risk-** A substitution at -308 |
| Oka et al. (60) | 2018 | Japan | 343 | 828 | **TNIP1** | **Risk-** C allele of rs7708392 |

***Polymorphism of the Fas gene at position 2670 does not influence susceptibility to AIH, but may affect the early development of cirrhosis.

**References cited only in the supplementary tables-**

40. Strettell MD, Donaldson PT, Thomson LJ, Santrach PJ, Moore SB, Czaja AJ, et al. Allelic basis for HLA-encoded susceptibility to type 1 autoimmune hepatitis. *Gastroenterology* (1997) 112:2028–35. doi: 10.1053/gast.1997.v112.pm9178696

41. Yoshizawa K, Ota M, Katsuyama Y, Ichijo T, Matsumoto A, Tanaka E, et al. Genetic analysis of the HLA region of Japanese patients with type 1 autoimmune hepatitis. J *Hepatol* (2005) 42:578–84. doi: 10.1016/j.jhep.2004.12.019

42. Umemura T, Katsuyama Y, Yoshizawa K, Kimura T, Joshita S, Komatsu M, et al. Human leukocyte antigen class II haplotypes affect clinical characteristics and progression of type 1 autoimmune hepatitis in Japan. *PLoS One* (2014) 9:e100565. doi:10.1371/journal.pone.0100565

43. Furumoto Y, Asano T, Sugita T, Abe H, Chuganji Y, Fujiki K, et al. Evaluation of the role of HLA-DR antigens in Japanese type 1 autoimmune hepatitis. BMC Gastroenterol (2015) 15:144. doi: 10.1186/s12876-015-0360-9

44. Oka S, Furukawa H, Yasunami M, Kawasaki A, Nakamura H, Nakamura M, et al. HLA-DRB1 and DQB1 alleles in Japanese type 1 autoimmune hepatitis: The predisposing role of the DR4/DR8 heterozygous genotype. *PLoS One* (2017) 12:e0187325. doi: 10.1371/ journal.pone.0187325

45. Bittencourt PL, Goldberg AC, Cançado EL, Porta G, Carrilho FJ, Farias AQ, et al. Genetic heterogeneity in susceptibility to autoimmune hepatitis types 1 and 2. Am J *Gastroenterol* (1999) 94:1906–13. doi: 10.1111/j.1572-0241.1999.01229.x

46. Duarte-Rey C, Pardo AL, Rodríguez-Velosa Y, Mantilla RD, Anaya JM, Rojas-Villarraga A. HLA class II association with autoimmune hepatitis in Latin America: a meta-analysis. *Autoimmun Rev* (2009) 8:325–31. doi: 10.1016/j.autrev.2008.11.005

47. Hassan N, Siddiqui AR, Abbas Z, Hassan SM, Soomro GB, Mubarak M, et al. Clinical Profile and HLA Typing of Autoimmune Hepatitis From Pakistan. *Hepat Mon* (2013) 13:e13598. doi: 10.5812/hepatmon.13598

48. Lim YS, Oh HB, Choi SE, Kwon OJ, Heo YS, Lee HC, et al. Susceptibility to type 1 autoimmune hepatitis is associated with shared amino acid sequences at positions 70-74 of the HLA-DRB1 molecule. *J Hepatol* (2008) 48:133–9. doi: 10.1016/j.jhep.2007.08.019

49. QIU, DE‐KAI, and Xiong Ma. "Relationship between human leukocyte antigen‐DRB1 and autoimmune hepatitis type I in Chinese patients. *J Gastroenterol Hepatol* (2003) 18: 63-67. doi: 10.1046/j.1440-1746.2003.02918.x

50. Webb G, Chen YY, Li KK, Neil D, Oo YH, Richter A, et al. Single-gene association between GATA-2 and autoimmune hepatitis: A novel genetic insight highlighting immunologic pathways to disease. *J Hepatol* (2016) 64:1190–3. doi: 10.1016/j.jhep.2016.01.017

51. Sun W, Wu HY, Chen S. Influence of TBX21 T-1993C variant on autoimmune hepatitis development by Yin-Yang 1 binding. *World J Gastroenterol* (2017) 23:8500–11. doi: 10.3748/wjg.v23.i48.8500

52. Umemura T, Joshita S, Hamano H, Yoshizawa K, Kawa S, Tanaka E, et al. Association of autoimmune hepatitis with Src homology 2 adaptor protein 3 gene polymorphisms in Japanese patients. *J Hum Genet* (2017) 62:963–7. doi: 10.1038/jhg.2017.74

53. Littera R, Chessa L, Onali S, Figorilli F, Lai S, Secci L, et al. Exploring the role of killer cell immunoglobulin-like receptors and their HLA class I ligands in autoimmune hepatitis. *PLoS One* (2016) 11:e0146086. doi: 10.1371/journal.pone.0146086

54. Agarwal K, Czaja AJ, Donaldson PT. A functional Fas promoter polymorphism is associated with a severe phenotype in type 1 autoimmune hepatitis characterized by early development of cirrhosis. *Tissue Antigens* (2007) 69:227–35. doi: 10.1111/j.1399-0039.2006.00794.x

55. Kempinska-Podhorodecka A, Adamowicz M, Chmielarz M, Janik MK, Milkiewicz P, Milkiewicz M. Vitamin-D Receptor-Gene Polymorphisms Affect Quality of Life in Patients with Autoimmune Liver Diseases. *Nutrients* (2020) 12:2244. doi: 10.3390/nu12082244

56. Chaouali M, Carvalho A, Tezeghdenti A, Ben Azaiez M, Cunha C, Ghazouani E, et al. Cytotoxic T lymphocyte antigen-4 gene polymorphisms and susceptibility to type 1 autoimmune hepatitis in the Tunisian population. *Genes Dis* (2018) 5:256–62. doi: 10.1016/j.gendis.2017.12.006

57. Higuchi T, Oka S, Furukawa H, Nakamura M, Komori A, Abiru S, et al. Association of a single nucleotide polymorphism upstream of ICOS with Japanese autoimmune hepatitis type 1. *J Hum Genet* (2017) 62:481–4. doi: 10.1038/jhg.2016.155

58. Assis DN, Leng L, Du X, Zhang CK, Grieb G, Merk M, et al. The role of macrophage migration inhibitory factor in autoimmune liver disease. *Hepatology* (2014) 59:580–91. doi: 10.1002/hep.26664

59. Czaja AJ, Cookson S, Constantini PK, Clare M, Underhill JA, Donaldson PT. Cytokine polymorphisms associated with clinical features and treatment outcome in type 1 autoimmune hepatitis. *Gastroenterology* (1999) 117:645–52. doi: 10.1016/s0016-5085(99)70458-0

60. Oka S, Higuchi T, Furukawa H, Nakamura M, Komori A, Abiru S, et al. Association of a single nucleotide polymorphism in TNIP1 with type-1 autoimmune hepatitis in the Japanese population. *J Hum Genet* (2018) 63:739–44. doi: 10.1038/s10038-018-0440-0.
