# Supplementary material for: HLA and Non-HLA gene polymorphisms in autoimmune hepatitis patients of North Indian adults
Source: Front Immunol. 2023 Jan 18;13:984083. doi: 10.3389/fimmu.2022.984083 (PMC9891307; doi:10.3389/fimmu.2022.984083)
Supplement: Supplementary file 2 [file DataSheet_1.pdf]

Supplementary File S1

1. **HLA typing protocol**

HLA DRB1 Typing was done by Luminex based reverse Sequence Specific Oligonucleotide Probing (SSOP). The DNA of cases and controls were dissolved in Tris buffer to get the recommended concentration of 70-80ng/μL. The ratio of OD260/OD280 was maintained at 1.8 ± 10%. The master mix (provided with the kit) was kept at room temperature (18-30°C). The master mix was vortexed for around 10 seconds. The PCR tubes were capped and kept in thermocycler and PCR program was run. The HLA DRB1 probe mix (provided with the kit) was warmed in dry bath at 55°C to 60°C for at least 5-10 minutes. The probe mix was then vortexed for 30 seconds. 5μL each of PCR products and 15μL of probe mix were combined in each well of coaster plate. The coaster plates were sealed with polyethylene plate and placed in thermocycler. The hybridisationprogramme was run. SAPE was diluted by combining 170 μL of diluent (provided with the kit) and 0.85 μL of SAPE (provided with the kit) for each sample. All the samples (kept at 56°C) were diluted with 170 μL of diluted SAPE. After diluting the samples, the plate was removed from the thermocycler. The samples were then acquired using Luminex Fluoroanalyzer and then results were analyzed using Match IT DNA.

2. **SNV genotyping for CTLA-4 and PTPN22 gene**

In simple probe-based SNiP arrays probes were labeled with only one fluorophore, i.e. fluorescein. The probes were designed to specifically hybridize to the target sequence that contained SNV of interest. Once hybridized to its target sequence, simple probes emitted more fluorescence than non-hybridized template. If the simple probe was free in solution, emission of the reporter dye was quenched by specific non-fluorescent quencher. When the probe hybridizes to its target, quenching was reduced and fluorescein emitted green fluorescence. Even when the probe was not hybridized, background fluorescence was detectable at 530 nm, so the signal to noise ratio was low. After amplification of the target sequence, the melting behavior of the simple probes was monitored. The more stable the hybridization between simple probe and target sequence (i.e. for the wild type), the higher the melting temperature. Variant allele weakened the stability of simple probe binding. Ninety-six wells plates coated with respective primers and probes were used. Reaction mixture was prepared as following: Sterile ddH_2O – 14.8 μl, $MgCl_2$ (25mM) - 1.2 μl, Genotyping master mix (5X) - 2 μl for single well. The reaction mixture was pipetted into the 96 wells and after that 2 μl of DNA (diluted appropriately to adjust the concentration ≈ 50 ng was added to the wells from A to H in the columns 1 - 12 in duplicates. The PCR was performed on LC 480 (Light cycler 480, Roche) following the program: Initial denaturation: 95℃ for 10 mins (ramp rate 4.4), 45 cycles 95℃ for 10s (ramp rate 4.4), 60℃ for 10s (ramp rate 2.2), 72℃ for 15s (ramp rate 4.4). Melting curve analysis was done by following the program: 95℃ for 30s (ramp rate 4.4), 35℃ for 2 mins (ramp rate 1.5) and 75℃ continuous followed by cooling at 40℃ for 30s (ramp rate 1.5). Analysis was done thereafter, by Tm calling. Negative derivative of fluorescence over-temperature gave distinct peaks for wild type, variant type and heterozygote type. The melting curves were then read for the respective Tm of the peaks obtained, and alleles were assigned accordingly.
